# Class 3 semaphorins negatively regulate dermal lymphatic network formation

**DOI:** 10.1242/bio.012302

**Published:** 2015-08-28

**Authors:** Yutaka Uchida, Jennifer M. James, Fumikazu Suto, Yoh-suke Mukouyama

**Affiliations:** 1 Laboratory of Stem Cell and Neuro-Vascular Biology, Genetics and Developmental Biology Center, National Heart, Lung, and Blood Institute, National Institutes of Health, Building 10/6C103, 10 Center Drive, Bethesda, MD 20892, USA; 2 Department of Ultrastructural Research, National Institute of Neuroscience, National Center of Neurology and Psychiatry, 4-1-1 Ogawahigashi, Kodaira, Tokyo 187-8502, Japan

**Keywords:** SEMA3s, NRP2, Dermal lymphangiogenesis, LEC sprouting, LEC growth

## Abstract

The development of a patterned lymphatic vascular network is essential for proper lymphatic functions during organ development and homeostasis. Here we report that class 3 semaphorins (SEMA3s), SEMA3F and SEMA3G negatively regulate lymphatic endothelial cell (LEC) growth and sprouting to control dermal lymphatic network formation. Neuropilin2 (NRP2) functions as a receptor for SEMA3F and SEMA3G, as well as vascular endothelial growth factor C (VEGFC). In culture, Both SEMA3F and SEMA3G inhibit VEGFC-mediated sprouting and proliferation of human dermal LECs. In the developing mouse skin, *Sema3f* is expressed in the epidermis and *Sema3g* expression is restricted to arteries, whereas their receptor *Nrp2* is preferentially expressed by lymphatic vessels. Both *Sema3f;Sema3g* double mutants and *Nrp2* mutants exhibit increased LEC growth in the skin. In contrast, *Sema3f;Sema3g* double mutants display increased lymphatic branching, while *Nrp2* mutants exhibit reduced lymphatic branching. A targeted mutation in *PlexinA1* or *PlexinA2*, signal transducers forming a receptor complex with NRP2 for SEMA3s, exhibits an increase in LEC growth and lymphatic branching as observed in *Sema3f;Sema3g* double mutants. Our results provide the first evidence that SEMA3F and SEMA3G function as a negative regulator for dermal lymphangiogenesis *in vivo*. The reciprocal phenotype in lymphatic branching between *Sema3f;Sema3g* double mutants and *Nrp2* mutants suggest a complex NRP2 function that regulates LEC behavior both positively and negatively, through a binding with VEGFC or SEMA3s.

## INTRODUCTION

Vascular endothelial growth factor C (VEGFC) and its receptor, VEGF receptor 3 (VEGFR3) are key signaling molecules required for lymphatic development (Dumont et al., 1998; Karkkainen et al., 2004; Makinen et al., 2001). VEGFC stimulates lymphatic endothelial cell (LEC) proliferation, survival, and migration *in vitro* and *in vivo* (Jeltsch et al., 1997; Makinen et al., 2001). In *Vegfc* mutant mice, LEC progenitors initially differentiate in the embryonic cardinal veins, but fail to migrate and form the primary lymph sacs, resulting in the complete absence of peripheral lymphatic vasculature (Karkkainen et al., 2004).

Neuropilin2 (NRP2) acts as a co-receptor to modulate VEGFC/VEGFR3 signaling in lymphatic vessel development. The interaction of NRP2 with VEGFR3 enhances VEGFC-induced LEC survival and migration *in vitro* (Favier et al., 2006; Gluzman-Poltorak et al., 2001). *Nrp2* mutants show a severe reduction of small-caliber lymphatic vessels (Yuan et al., 2002). Further genetic analysis indicates that a VEGFR3/NRP2 receptor complex mediates VEGFC-induced lymphatic sprouting (Xu et al., 2010). Selective disruption of VEGFC binding to NRP2 by an anti-NRP2 blocking antibody also leads to the reduction of LEC sprouting but does not inhibit LEC proliferation (Caunt et al., 2008).

NRP2 also functions as a co-receptor with a signal transducer PlexinA for class 3 semaphorins (SEMA3s), a family of ligands first identified for its role in axon guidance (Giger et al., 1998). SEMA3B and SEMA3C can bind to NRP2 as well as NRP1, whereas SEMA3F and SEMA3G have a higher binding affinity for NRP2 (Chen et al., 2000; Giger et al., 1998; Takahashi et al., 1998; Taniguchi et al., 2005). In addition to their role as axonal guidance cues (Pasterkamp and Kolodkin, 2003), SEMA3s are involved in developmental angiogenesis, tumor angiogenesis, lymphatic valve formation and organ development. For example, SEMA3F can act as a functional inhibitor of tumor cell growth, metastasis and tumor angiogenesis (reviewed in Bielenberg et al., 2006). SEMA3F also inhibits VEGFC or VEGFA-mediated endothelial cell migration and proliferation *in vitro* (Bielenberg et al., 2004; Favier et al., 2006; Kessler et al., 2004). SEMA3F and/or SEMA3G signal through NRP2 controls neural crest cell migration (Gammill et al., 2006; Yu and Moens, 2005). Recently, it has been reported that SEMA3A/PlexinA1/NRP1 signaling is important for lymphatic valves formation (Bouvree et al., 2012; Jurisic et al., 2012). Although the VEGFR3/NRP2 complex is important for VEGFC-mediated LEC sprouting, whether SEMA3/PlexinA/NRP2 signaling is also involved in lymphatic development remains unclear.

In this study, we provide the first evidence demonstrating that SEMA3F and SEMA3G negatively regulate lymphatic vessel development. The NRP2 receptor has a dual function that regulates lymphatic sprouting and LEC proliferation both positively and negatively, through a binding with VEGFC or SEMA3s.

## RESULTS

### SEMA3F and SEMA3G inhibit LEC sprouting and proliferation in culture

**Fig. 1.**
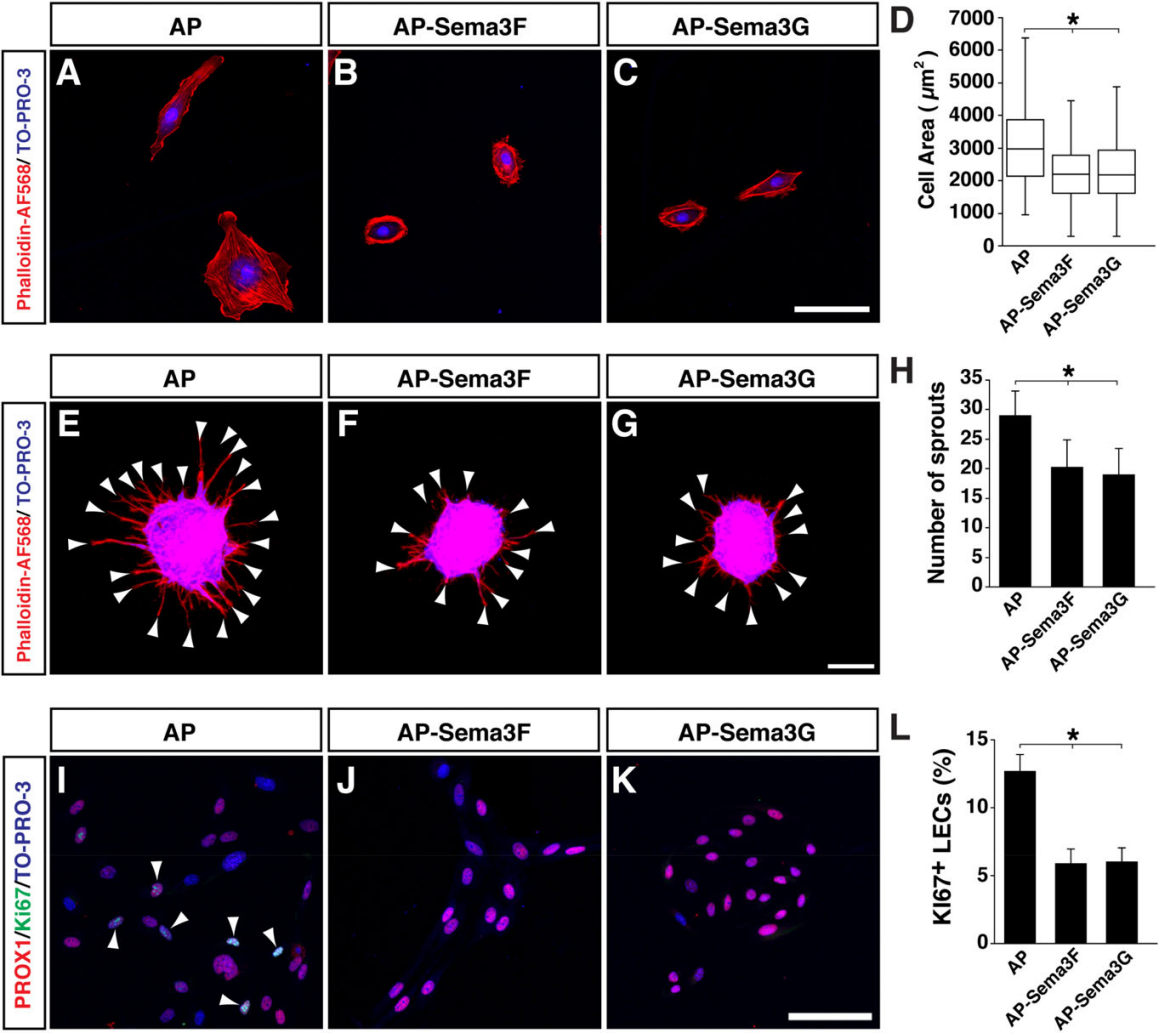
**SEMA3F and SEMA3G induce LEC contraction and inhibit LEC sprouting in culture.** (A-C) Cell contraction assay for human LECs with 1 nM of AP control protein (A), AP-SEMA3F(R580A/R582A) (B) or AP-SEMA3G (C). Cell shape is visualized by Phalloidin-AF568 staining and nuclei are stained by the nuclear marker TO-PRO-3. (D) Quantification of total cell area. *N*=231 cells, AP control; *N*=278, AP-SEMA3F; *N*=260, AP-SEMA3G; two independent experiments. (E-G) Sprouting assay for human LECs in response to VEGFC with 0.5 nM AP (E), VEGFC with 1 nM AP-SEMA3F (R580A/R582A) (F), or VEGFC with 0.5 nM AP-SEMA3G (G). Arrowheads indicate each sprout. Cell sprouts are visualized by Phalloidin-AF568 staining and nuclei are stained by the nuclear marker TO-PRO-3. (H) Quantification of total number of LEC sprouts per one aggregate. *N*=8 from two independent experiments, bars represent mean±s.d. (I-K) Proliferation assay for human LECs in response to VEGFC with 0.5 nM AP protein (I), VEGFC with 0.5 nM AP-SEMA3F(R580A/R582A) (J), or VEGFC with 0.5 nM AP-SEMA3G (K). The cells were stained with the LEC marker PROX1 (red), proliferation marker Ki67 (green) and TO-PRO-3 (blue). Arrowheads indicate PROX1 and Ki67 double positive cells. (L) Quantification of Ki67^+^/PROX1^+^ LECs. *N*=43 images, AP control; *N*=34, AP-SEMA3F; *N*=39, AP-SEMA3G; from 2-3 independent experiments were analyzed. Bars represent mean±s.e.m. *P<0.01, one-way ANOVA with Tukey-HSD multiple comparison test. Scale bars: 100 µm.

To examine how lymphatic endothelial cells (LECs) respond to SEMA3F and SEMA3G in culture, dermal-derived human lymphatic microvascular ECs (HMVEC-dLy-Neo; human LECs) were treated with AP-SEMA3F and AP-SEMA3G. Both SEMA3F and SEMA3G dramatically induced cell contraction (Fig. 1A-D). We next examined whether SEMA3F and SEMA3G influence VEGFC-mediated LEC behavior *in vitro*. VEGFC stimulates sprouting and proliferation of human LECs in a dose-dependent manner (data not shown). We found that both SEMA3F and SEMA3G inhibit VEGFC-mediated human LEC sprouting in 3D collagen gel (Fig. 1E-H). We also found that both SEMA3F and SEMA3G inhibit VEGFC-induced human LEC proliferation (Fig. 1I-L). These results demonstrate that SEMA3F and SEMA3G inhibit VEGFC-mediated human LEC sprouting and proliferation.

### Differential expression patterns of SEMA3s in the embryonic skin

**Fig. 2.**
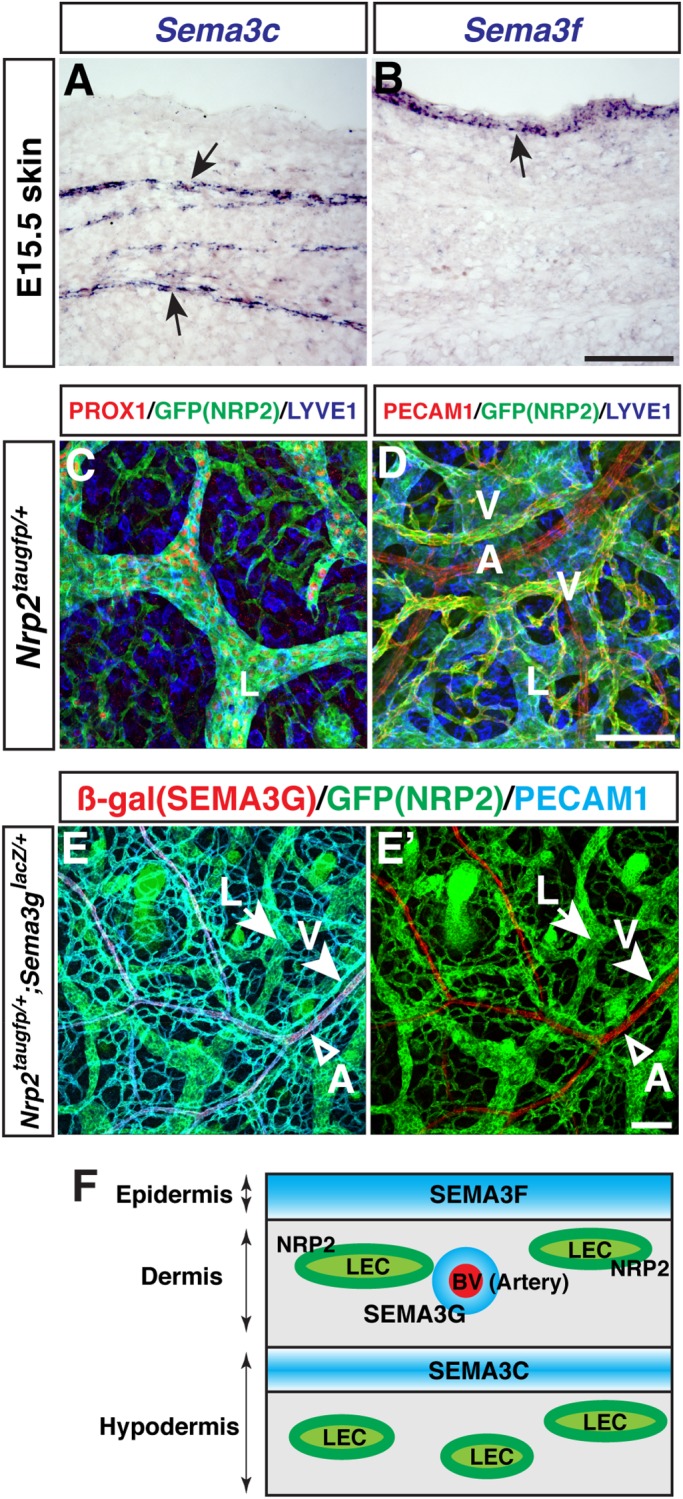
**Expression of SEMA3s and their receptor NRP2 in limb skin.** (A,B) Expression of *Sema3c* and *Sema3f* in E15.5 skin (arrows). *In situ* hybridization on serial transverse sections was performed with the indicated probes. (C,D) Whole-mount limb skin staining of E17.5 *Nrp2^taugfp/+^* embryos with antibodies to the LEC marker PROX1 (red), LYVE1 (blue), and the EC marker PECAM1 (red) together with GFP (*Nrp2^taugfp^*, green). A, arteries; L, lymphatic vessels; V, veins. (E,E′) Whole-mount limb skin staining of E17.5 *Sema3g^lacZ/+^;Nrp2^taugfp/+^* embryos with antibodies to β-gal (*Sema3g^lacZ^*, red), GFP (*Nrp2^taugfp^*, green), and PECAM1 (blue). *Sema3g^lacZ^* is expressed in arteries (A, open arrowhead), whereas *Nrp2^taugfp^* is expressed in both lymphatic vessels (L, arrow) and veins (V, arrowhead). (F) Schematic model illustrating differential expression patterns of SEMA3s and NRP2 in the skin. Scale bars: 100 µm.

We next examined the expression patterns of *Sema3b*, *Sema3c*, *Sema3f* and *Sema3g* in E15.5 skin by *in situ* hybridization, stages when lymphatic network is actively developing in the skin. We found distinct expression patterns of *Sema3c* and *Sema3f* (Fig. 2A,B), albeit a less clear *in situ* hybridization signal for *Sema3b* and *Sema3g* (data not shown). Expression of *Sema3c* was detectable in the layer adjacent to the boundary between the dermis and hypodermis (Fig. 2A). Consistent with a previous report (Eckhardt and Meyerhans, 1998), expression of *Sema3f* was observed in the epidermis (Fig. 2B). Although the *Sema3g in situ* hybridization signal was not clearly detectable, the expression of SEMA3G using *Sema3g^lacZ/+^* heterozygous embryos, carrying a *lacZ* reporter cassette under the endogenous *Sema3g* promoter, was restricted to arteries and is not expressed in veins or lymphatic vessels within the skin (Fig. 2E,E′; Kutschera et al., 2011).

These SEMA3s are known to exert repulsive functions by activating their cognate NRP receptors. Examination of NRP2 expression using *Nrp2^taugfp/+^* heterozygous embryos, carrying a *tauGFP* reporter cassette under the endogenous *Nrp2* promoter (Walz et al., 2002), revealed that *Nrp2^taugfp^* was consistently detected in LECs expressing the LEC markers PROX1 and LYVE1 (Fig. 2C) and in venous ECs expressing the pan-EC marker PECAM1 (Fig. 2D). Interestingly, we discovered mutually exclusive expression of *Nrp2^taugfp^* and *Sema3g^lacZ^* in the double heterozygous embryos (Fig. 2E,E′). The unique expression patterns of SEMA3s adjacent to NRP2-expressing LECs suggest that multiple SEMA3s may affect lymphatic network formation in the skin (Fig. 2F).

### Abnormal lymphatic vessel network formation in *Sema3s* and *Nrp2* mutants

**Fig. 3.**
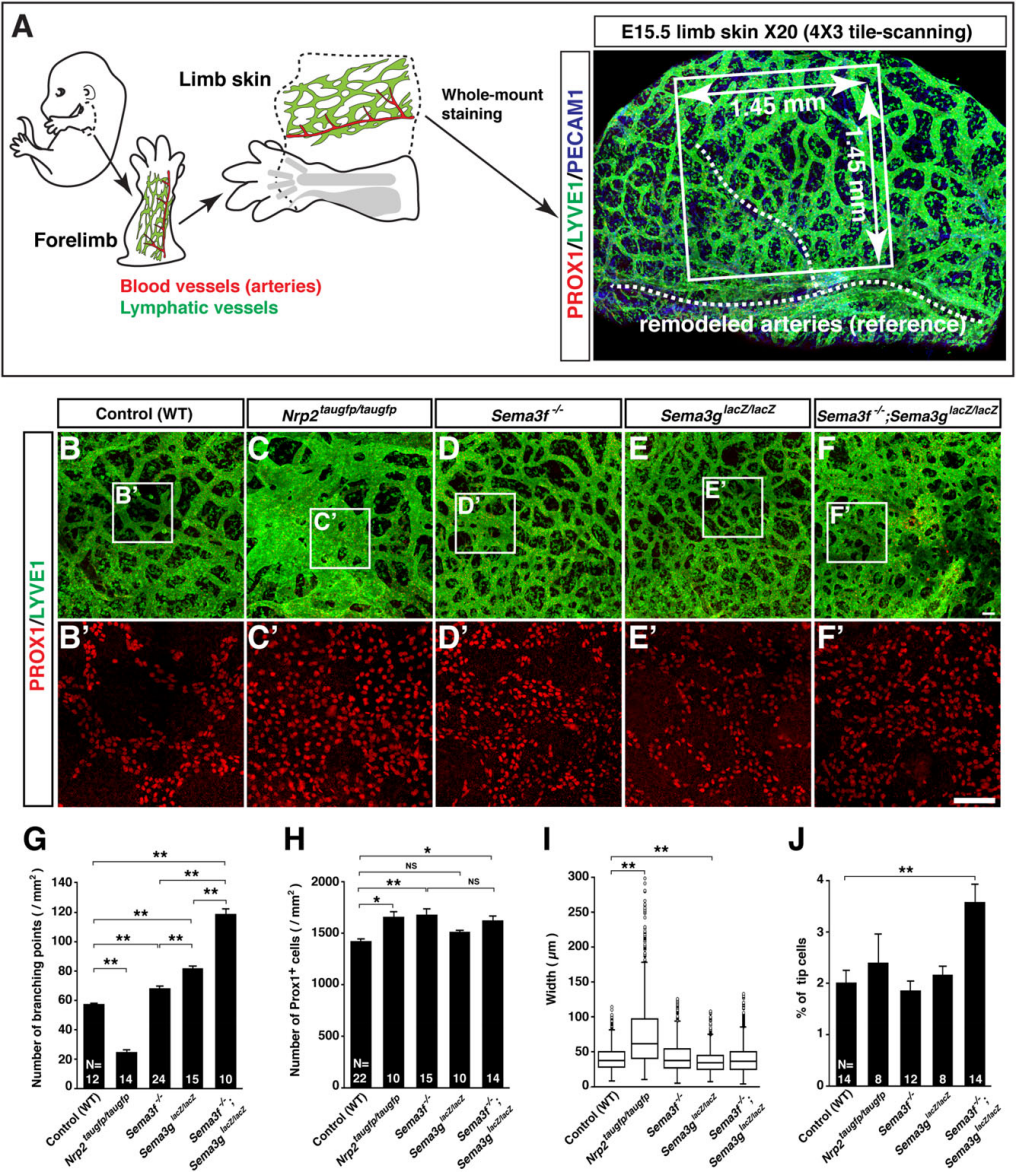
**SEMA3-NRP2 signaling is required for lymphatic network formation in limb skin.** (A) Schematic diagram illustrating forelimb skin dissection and whole-mount limb skin staining for analysis of lymphatic network formation. The forelimb is dissected at the base of shoulder (dashed line) from E15.5 embryos, and then limb skin is peeled off along the dashed line. Whole-mount immunolabeling of limb skin is performed with antibodies to the LEC markers PROX1 and LYVE1, and the pan-endothelial cell markers PECAM1. For multiple quantification measurements, we defined an image area (white box, 1.45 mm×1.45 mm) in 20× confocal tiled z-stack images using the position of large-diameter blood vessels (white dashed line) as a frame of reference. The PROX1 staining visualizes nuclei of LECs that allows us to measure LEC number in the lymphatic vasculature. The LYVE1 staining allows us to measure lymphatic branching points. Note that the LYVE1 staining also detects tissue macrophages in the skin. (B-F′) Whole-mount staining of limb skin from E15.5 mutants and wild-type (WT) controls with PROX1 (red) and LYVE1 (green). The boxed regions in (B-F) are magnified in (B′-F′), respectively: The magnified images show PROX1 only. Scale bars: 100 µm. (G-J) Quantification of lymphatic branching points (G) and total LEC number (H) per area (mm^2^). Lymphatic vessel width at the middle of lymphatic branching points represented as box and whisker plot (I) (*N*=1404, WT controls; *N*=980, *Nrp2* mutants; *N*=1856, *Sema3f* mutants; *N*=2388, *Sema3g* mutants; *N*=3318, *Sema3f;Sema3g* double mutants). Quantification of lymphatic tip cells in total LECs (J). Bars represent mean±s.e.m. and sample numbers (the number of limb skins we analyzed) are shown in the bars. *P<0.05; **P<0.01; NS, not significant by one-way ANOVA with Tukey-HSD multiple comparison test.

To examine whether mutants lacking SEMA3s-mediated signaling exhibit defective lymphatic network formation in the embryonic skin, we first established a whole-mount imaging of embryonic limb skin lymphatic vasculature with quantification measurements (Fig. 3A). Using this method, we examined what happens to dermal lymphatic vessel development in mutants lacking *Nrp2*, *Sema3f* or *Sema3g*. Interestingly, *Nrp2^taugfp/taugfp^*, *Sema3f^−/−^*, *Sema3g^lacZ/lacZ^* or *Sema3f^−/−^;Sema3g^lacZ/lacZ^* double mutants showed different phenotypes in lymphatic branching morphogenesis and LEC growth (Fig. 3B-F). Analysis of branching point phenotypes revealed that *Nrp2^taugfp/taugfp^* mutants exhibited decreased lymphatic branching complexity (Fig. 3B vs C; G). In contrast, both *Sema3f^−/−^* and *Sema3g^lacZ/lacZ^* mutants exhibited increased lymphatic branching complexity, albeit more branching in *Sema3g^lacZ/lacZ^* mutants than *Sema3f^−/−^* mutants (Fig. 3B vs D and E; G). Furthermore, *Sema3f^−/−^;Sema3g^lacZ/lacZ^* double mutants displayed a synergistic increase in lymphatic branching points (Fig. 3B vs D vs E vs F; G), which indicates both SEMA3F and SEMA3G cooperatively inhibit lymphatic sprouting in *in vivo*. Quantification analysis of PROX1^+^ LEC number revealed that *Nrp2^taugfp/taugfp^* mutants exhibited increased LEC number resulting in lymphatic hyperplasia (Fig. 3B′ vs C′; H). Like *Nrp2^taugfp/taugfp^* mutants, *Sema3f^−/−^* mutants, but not *Sema3g^lacZ/lacZ^* mutants*,* exhibited a significant increase in LEC number (Fig. 3B′ vs D′ vs E′; H). Interestingly, *Sema3f^−/−^;Sema3g^lacZ/lacZ^* double mutants exhibited increased PROX1^+^ LEC number, but not a synergistic increase (Fig. 3B′ vs D′ vs E′ vs F′; H), almost similar number with *Sema3f^−/−^* mutants. This result suggests that SEMA3F but not SEMA3G is responsible to control PROX1^+^ LEC number *in vivo*. Furthermore, *Nrp2^taugfp/taugfp^* mutants exhibited an increase lymphatic width whereas *Sema3g^lacZ/lacZ^* had a decrease width (Fig. 3B vs C vs E; I). The increased branching complexity of *Sema3f^−/−^;Sema3g^lacZ/lacZ^* double mutants may be due to an increased tip cell formation (Fig. 3J).

### SEMA3F negatively regulate LEC proliferation in the skin

**Fig. 4.**
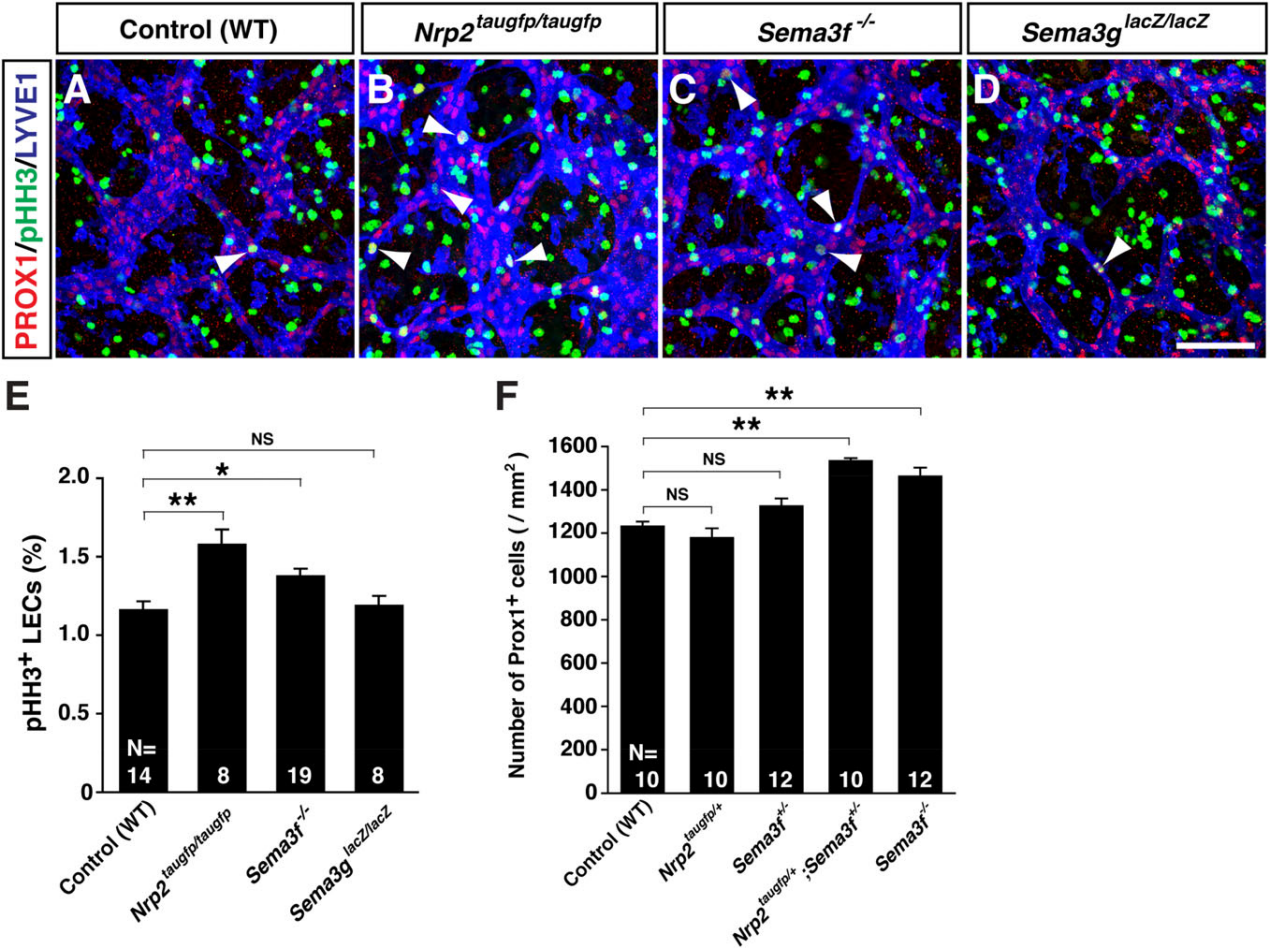
**SEMA3F-NRP2 signaling is responsible for inhibition of LEC proliferation in limb skin.** (A-D) Whole-mount limb skin staining of E14.5 mutants and WT controls with the proliferation marker phosphohistone H3 (pHH3, green) in addition to PROX1 (red) and LYVE1 (blue). Arrowheads indicate pHH3 positive proliferating LECs. Scale bar: 100 µm. (E) Quantification of pHH3^+^/PROX1^+^ LECs at E14.5 limb skin. (F) Quantification in genetic interaction analysis of Prox1^+^ LEC number per area (mm^2^) in E15.5 limb skin between *Nrp2* and *Sema3f* mutants. Bars represent mean±s.e.m. and sample numbers (the number of limb skins we analyzed) are shown in the bars. *P<0.05 and **P<0.01; NS, not significant by one-way ANOVA with Tukey-HSD multiple comparison test.

We next examined whether the increased LEC number results from an increased LEC proliferation in the mutants. LEC proliferation at E14.5 was significantly increased in *Nrp2^taugfp/taugfp^* mutants and *Sema3f^−/−^* mutants, compared to *Sema3g^lacZ/lacZ^* mutants and WT controls (Fig. 4A-E).

We further examined whether SEMA3F inhibits LEC proliferation through NRP2 *in vivo*. To address this question, we pursued the genetic interaction experiments in the LEC number between *Sema3f* and *Nrp2*. There was a significant increase in the PROX1^+^ LEC number in *Nrp2^taugfp/+^;Sema3f^+/−^* double heterozygous mutants compared with WT controls, *Nrp2^taugfp/+^* heterozygous, or *Sema3f^+/−^* heterozygous mutants (Fig. 4F). The increase of LEC number in *Nrp2^taugfp/+^;Sema3f^+/−^* double heterozygous mutants was as much as that in *Sema3f^−/−^* homozygous mutants which indicate a clear genetic interaction in the LEC number between *Sema3f* and *Nrp2.* These data suggest that SEMA3F inhibits LEC proliferation through NRP2.

### *PlexinAs* mutants display abnormal lymphatic vessel development as observed in *Sema3s* mutants

**Fig. 5.**
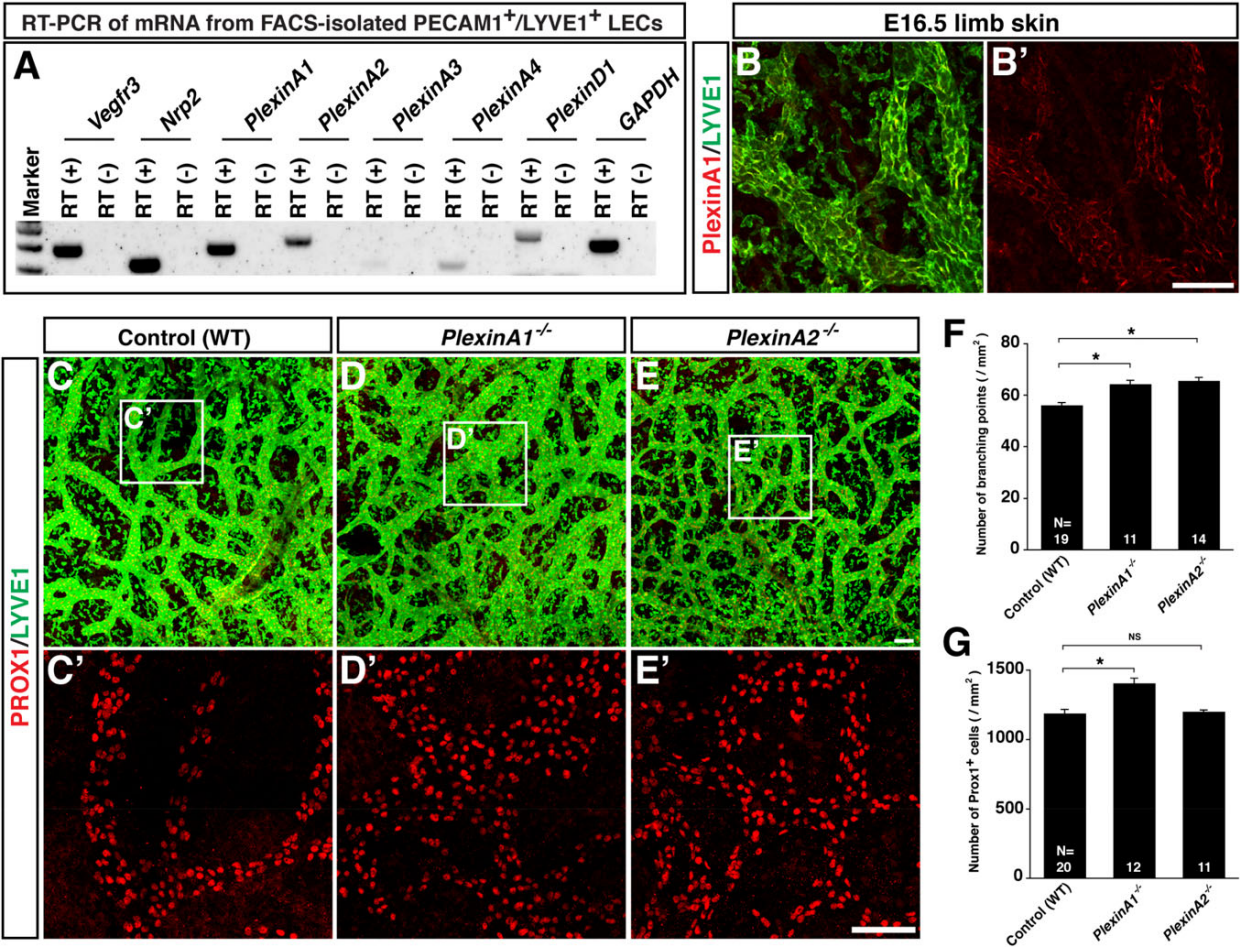
**SEMA3-PlexinA signaling is required for lymphatic network formation in limb skin.** (A) RT-PCR analysis of mRNA from FACS-isolated PECAM1^+^/LYVE1^+^ LECs from E16.5 embryos. (B) Whole-mount staining of E16.5 limb skin with antibodies to PlexinA1 (red) together with LYVE1 (green). (C-E′) Whole-mount staining of limb skins from E15.5 *PlexinA1*, *PlexinA2* mutants and WT controls with PROX1 (red) and LYVE1 (green). The boxed regions in (C-E) are magnified in (C′-E′), respectively: The magnified images show PROX1 only (C′-E′). Scale bars: 100 µm. (F,G) Quantification of lymphatic branching points (F) and total LEC number (G) per area (mm^2^). Bars represent mean±s.e.m. and sample numbers (the number of limb skins we analyzed) are shown in the bars. *P<0.01; NS, not significant by one-way ANOVA with Tukey-HSD multiple comparison test.

The increased branching in *Sema3f* and *Sema3g* mutants are opposite to what we expected based on our finding of decreased branching in *Nrp2* mutants (Fig. 3G). The phenotypic differences between *Sema3f/g double* mutants and *Nrp2* mutants might reflect a dual function of NRP2 that regulates lymphatic sprouting and LEC proliferation both positively and negatively, through a binding with VEGFC or SEMA3s. To further address the role of SEMA3s-mediated signaling, we decided to examine whether loss of PlexinA, a signal transducer and receptor complex with NRP2 for SEMA3s, influences lymphatic branching and LEC number. Among PlexinA family members, *PlexinA1* and *PlexinA2* are mainly expressed by FACS-isolated LECs (Fig. 5A). We further confirmed PlexinA1 expression in dermal lymphatic vasculature by anti-PlexinA1 specific antibody (Fig. 5B). *PlexinA1^−/−^* mutants exhibited an increase in lymphatic branching points and LEC number (Fig. 5C vs D; C′ vs D′; F,G), whereas *PlexinA2^−/−^* mutants exhibited only an increase in lymphatic branching points (Fig. 4C vs E; C′ vs E′; F,G). Interestingly, *PlexinA1^−/−^* mutants exhibit almost identical, albeit milder, phenotypes with *Sema3f^−/−^;Sema3g^lacZ/lacZ^* double mutants. These observations support that PlexinA functions as a physiological receptor for SEMA3F and SEMA3G *in vivo*.

### *Nrp2* knockdown diminishes SEMA3G-mediated inhibition of VEGFC-induced LEC sprouting in culture

The abovementioned results suggest that Sema3F/PlexinA1/NRP2 signaling controls LEC growth in lymphatic vessel development. However, phenotypic differences in lymphatic vessel branching between *Sema3f/g double* mutants and *Nrp2* mutants make it difficult to determine the role of Sema3s/NRP2 signaling in lymphatic branching. Indeed, there was not a clear genetic interaction experiments in lymphatic branching between *Nrp2* and *Sema3s* mutant mice (data not shown). To clarify the role of NRP2 on the LEC behavior, we therefore turned to *in vitro* LEC sprouting assay and examine what happens to SEMA3s-mediated inhibition of LEC sprouting in the absence of NRP2.

**Fig. 6.**
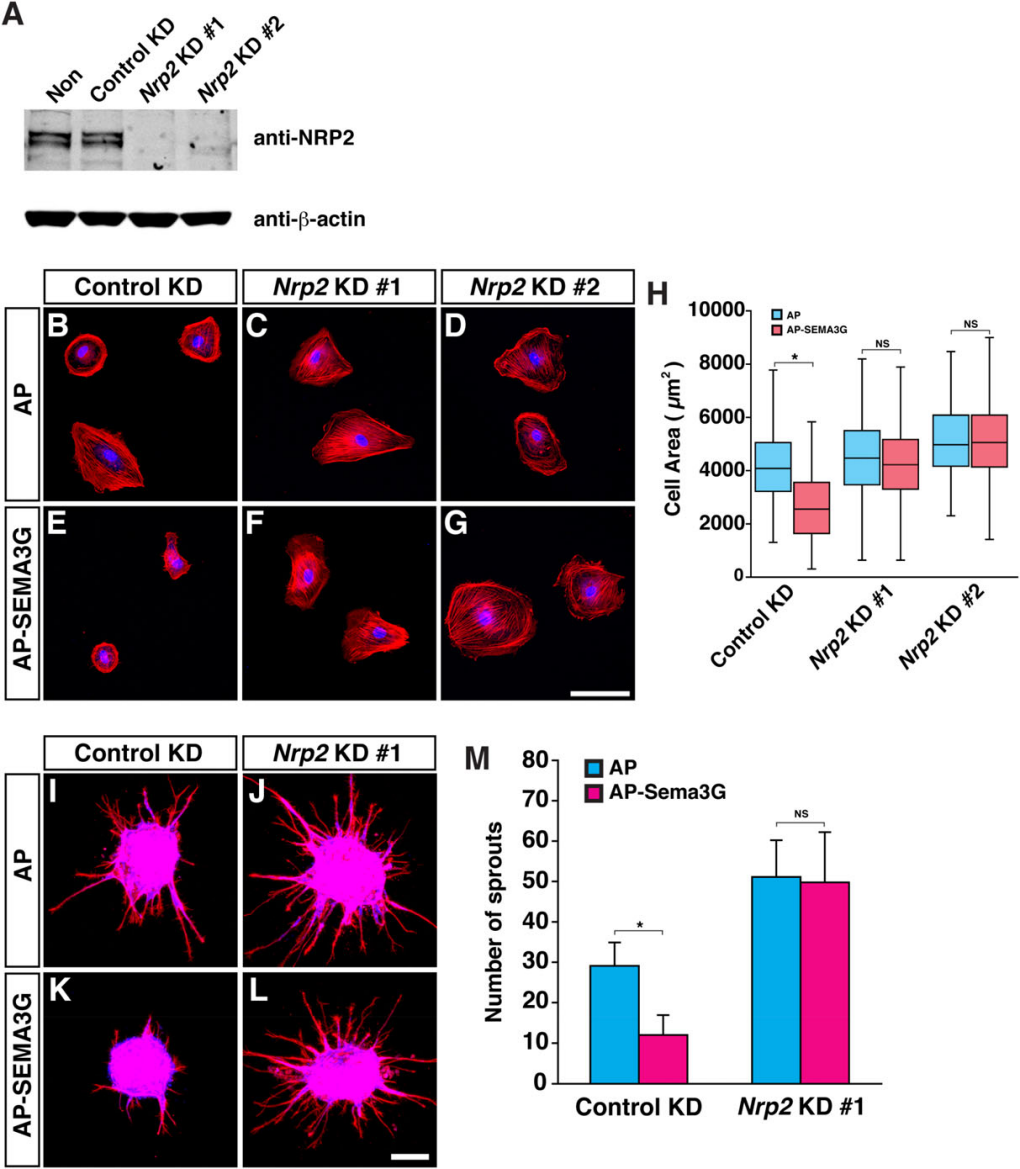
***Nrp2* deficient LECs fail to respond to Sema3G in culture.** (A) Immunoblot analysis probed for NRP2 and β-actin of control or *Nrp2* knockdown (KD) human LECs (HMVEC-dLy-Neo). (B-G) Cell contraction assay for control or *Nrp2* knockdown human LECs with 1 nM of AP control protein (B-D) or AP-SEMA3G (E-G). Phalloidin-AF568 staining shows cell shape and nuclei are stained by TO-PRO-3. (H) Quantification of total cell area in the cell contraction assay. Control KD LECs treated with AP control, *N*=263 cells; control KD LECs treated with AP-SEMA3G, *N*=296; *Nrp2* KD #1 LECs treated with AP control, *N*=187 cells; *Nrp2* KD #1 LECs treated with AP-SEMA3G; *N*=221; *Nrp2* KD #2 LECs treated with AP control, *N*=227; *Nrp2* KD #2 LECs treated with AP-SEMA3G, *N*=223; two independent experiments). (I-L) Sprouting assay for control or *Nrp2* knockdown human LECs in response to VEGFC with 0.5 nM of AP control protein (I,J) or AP-SEMA3G (K,L). Phalloidin-AF568 staining shows cell shape and nuclei are stained by TO-PRO-3. (M) Quantification of total number of LEC sprouts per one aggregate. (*N*=8 from two independent experiments, bars represent mean±s.d.). *P<0.01; NS, not significant by one-way ANOVA with Tukey-HSD multiple comparison test.

Knockdown of NRP2 expression in LECs was successfully carried out in culture (Fig. 6A). *Nrp2*-deficient LECs failed to respond to SEMA3G such that the cells did not show SEMA3G-induced cell contraction (Fig. 6B-H). Furthermore, the *Nrp2* deficiency diminished SEMA3G-mediated inhibition of VEGFC-induced LEC sprouting, although *Nrp2*-deficient LECs sprout more than control LECs (Fig. 6I-M). These results suggest that NRP2 is essential for SEMA3G-mediated inhibition of lymphatic sprouting.

### Lymphatic defects in the trunk and skin of *Sema3s* and *Nrp2* mutants at different developmental stages and adult

**Fig. 7.**
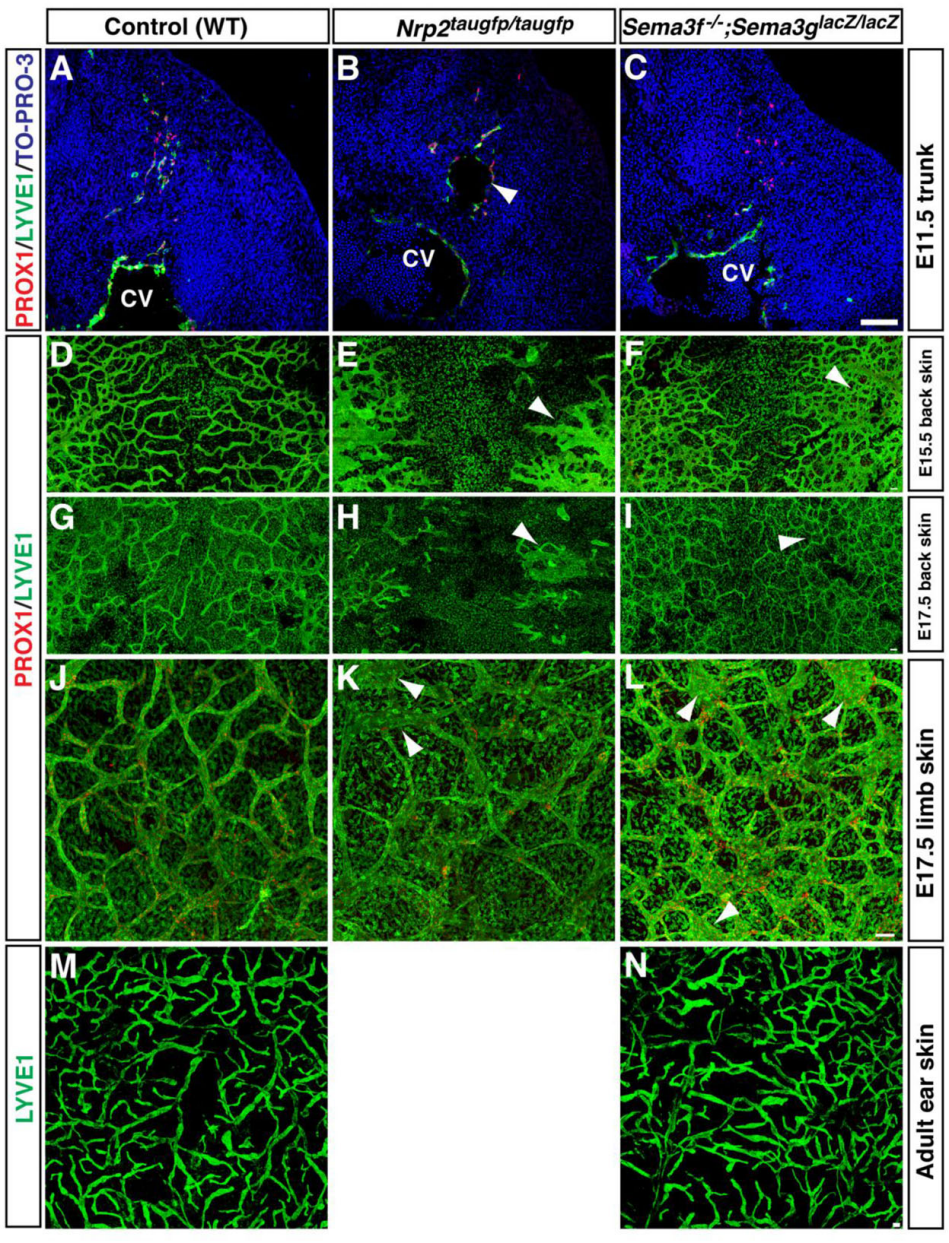
**Early and late stage phenotypes of *Nrp2* mutants and *Sema3f;Sema3g* double mutants.** (A-C) Section staining of E11.5 *Nrp2^taugfp/taugfp^* mutants and *Sema3f^−/−^;Sema3g^lacZ/lacZ^* double mutants and WT controls at trunk level with antibodies to PROX1 (red) and LYVE1 (green) together with the nuclear marker TO-PRO-3 (blue). *Nrp2* mutants exhibit lymph sac-like tubes, which remain relatively adjacent to the cardinal vein (arrowhead). CV, cardinal vein. (D-I) Whole-mount staining of back skin from E15.5 (D-F) and E17.5 (G-I) mutants and WT controls with antibodies to PROX1 (red) and LYVE1 (green). *Nrp2* mutants exhibit defective lymphatic vessels (arrowhead). *Sema3f;Sema3g* double mutants exhibit increased lymphatic branching points (arrowhead). (J-L) Whole-mount staining of limb skins from E17.5 mutants and WT controls with antibodies to PROX1 (red) and LYVE1 (green). *Nrp2* mutants and *Sema3f;Sema3g* double mutants exhibit enlarged lymphatic vessels (arrowhead). However, the defects in the mutant lymphatic vasculature at E17.5 are milder than that at E15.5 (compared to Fig. 3). (M,N) Whole-mount staining of adult ear skins from *Sema3f;Sema3g* double mutants and WT controls with an anti-LYVE1 antibody (green). Note that *Nrp2* mutants die after weaning. Scale bars: 100 µm.

**Fig. 8.**
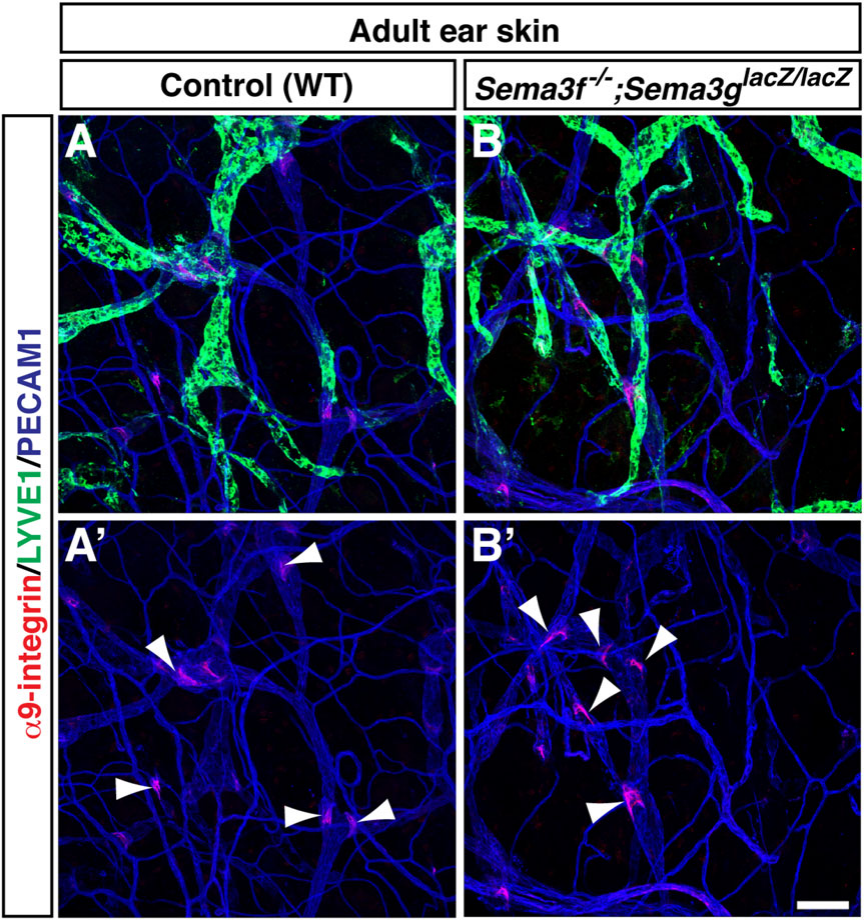
**Normal lymphatic valve formation in *Sema3f;Sema3g* double mutants.** Whole-mount staining of adult ear skin *Sema3f^−/−^;Sema3g^lacZ/lacZ^* double mutants (A,A′) and WT controls (B,B′) with antibodies to the lymphatic valve marker α9-integrin (red) together with LYVE1 (green) and an pan-endothelial cells marker PECAM1 (blue). Arrowheads indicate lymphatic valves. A′ and B′ show only red and blue channels of A and B, respectively. Scale bar: 100 µm.

Whether the SEMA3s-mediated negative regulation influences LEC progenitor at an earlier stage and lymphatic network maturation and maintenance in a later embryonic stage and adult remain to be determined. At E11.5, Prox1^+^ LEC progenitors bud from the anterior cardinal vein and migrate towards the superficial skin (Fig. 7A). No apparent defective LEC progenitor migration was observed in *Sema3f^−/−^;Sema3g^lacZ/lacZ^* double mutants, given that *Nrp2* mutants exhibit lymph sac-like tubes which remain relatively adjacent to the cardinal vein (Fig. 7B,C). Like the abovementioned lymphatic phenotypes in the limb skin, the back skin of *Nrp2* mutants exhibited hyperplastic lymphatic vasculature at E15.5 (Fig. 7E) and continued to display defective lymphatic vasculature at E17.5 (Fig. 7I). The back skin of *Sema3f^−/−^;Sema3g^lacZ/lacZ^* double mutants also exhibited hyper branching phenotype (Fig. 7F,I). Defective lymphatic network was found in E17.5 limb skin of *Nrp2* mutants and *Sema3f^−/−^;Sema3g^lacZ/lacZ^* double mutants (arrowhead in Fig. 7K,L). Consistent with the previous study (Yuan et al., 2002), however, the defective lymphatic network appears to be recovered from E17.5 onwards. Furthermore, no apparent defective lymphatic network and valve formation was detectable in the adult ear skin (Fig. 7N,M; Fig. 8A,B). These data suggest that the recovery of lymphatic vessel development occurred during later embryonic stages and postnatal life in the mutants.

## DISCUSSION

In this study, we provide the first genetic evidence that SEMA3F and SEMA3G negatively regulate both lymphatic sprouting and LEC proliferation to control lymphatic vessel network formation. SEMA3F and SEMA3G inhibit VEGFC mediated LEC sprouting and proliferation. Genetic inactivation of SEMA3s-mediated signaling leads to abnormal lymphatic branching network and LEC proliferation in the skin. Our findings define a key negative regulator that shapes the pattern of lymphatic vessel network formation.

Despite the significance of SEMA3s action on LEC growth through NRP2, the reciprocal phenotype in lymphatic branching complexity between *Nrp2^taugfp/taugfp^* homozygous mutants and *Sema3f^−/−^;Sema3g^lacZ/lacZ^* double homozygous mutants makes it challenging to definitely determine if NRP2 is required for SEMA3s-mediated negative regulation of lymphatic branching complexity: our genetic interaction experiments between *Sema3g* and *Nrp2* did not provide a clear answer for the phenotypic differences: *Sema3g^lacZ/+^* heterozygous mutants exhibited more branching points than *Nrp2^taugfp/+^* heterozygous and WT controls, whereas this increase is diminished in *Nrp2^taugfp/+^;Sema3g^lacZ/+^* double heterozygous mutants (data not shown). One possible explanation is that the reduction of lymphatic sprouting in *Nrp2* mutants may reflect the lack of VEGFC/VEGFR3/NRP2-mediated positive signaling. This possibility is supported by previous studies demonstrating that selectively inhibiting VEGFC binding to NRP2 with a function-blocking antibody results in the reduction of VEGFC-induced LEC migration but not LEC proliferation (Caunt et al., 2008) and further genetic analysis that a VEGFR3/NRP2 receptor complex mediates VEGFC-induced lymphatic sprouting (Xu et al., 2010). In addition, SEMA3F inhibits VEGFA and VEGFC-induced endothelial cell proliferation, survival and migration (Favier et al., 2006; Kessler et al., 2004). These studies suggest VEGFC/VEGFR3/NRP2 complex is necessary for lymphatic sprouting *in vivo*. *Nrp2* deficiency, which results in no VEGFC-induced positive sprouting signal, overrides the increase in branching complexity induced by the lack of SEMA3s/PlexinA/NRP2-mediated inhibitory signaling. The other possible explanation is that SEMA3s action on lymphatic sprouting might be NRP2-independent, although it depends on PlexinAs. It should be noted that NRP2 is known to interact with TGFβ signaling (Grandclement et al., 2011), HGF signaling (Sulpice et al., 2008) and/or Integrins signaling (Ou et al., 2015; Cao et al., 2013; Goel et al., 2013). These signaling pathways might affect lymphatic branching. Indeed, we have previously demonstrated that TGFβ signaling is required for sprouting lymphangiogenesis in the skin (James et al., 2013). Resolution of this issue, as well as direct demonstration that NRP2 is required for SEMA3s action on lymphatic sprouting *in vivo*, will require *Nrp2* mutant mice expressing *Nrp2* with a mutation which selectively abolishes SEMA3s-NRP2 binding.

Although SEMA3F and SEMA3G exhibit the same effects for human LECs in *in vitro* experiment which both inhibit sprouting and proliferation, we find different phenotypes in lymphatic branching complexity and LEC growth between *Sema3f* and *Sema3g* mutants. This observations may reflect the different expression patterns of SEMA3F and SEMA3G: Epidermis-derived SEMA3F globally influences LEC proliferation and sprouting at relatively low concentration, whereas arterial SEMA3G provides a local signal, at relatively high concentration, to inhibit lymphatic sprouting. Alternatively, SEMA3F and SEMA3G may bind to the PlexinAs/NRP2 receptor complexes with different binding affinities or different receptor complexes. Our observation that 0.5 nM of SEMA3G inhibits VEGFC-induced LEC sprouting, while 1 nM of SEMA3F inhibits the LEC sprouting (Fig. 1E-H) suggest that SEMA3s may have different binding affinity to distinct receptor complexes. Indeed, SEMA3F signaling functions through NRP2/PlexinA3 or NRP2/PlexinA1 complex but not NRP2/PlexinA2 complex (Doci et al., 2015). We also found that both *Sema3f* mutant and *PlexinA1* mutants exhibit an increased lymphatic branching and LEC number, while both *Sema3g* mutant and *PlexinA2* mutants exhibit an increased lymphatic branching. These results suggest that, at least in part, SEMA3G may preferentially function through PlexinA2 in lymphatic branching. Resolution of this issue *in vivo* will require a genetic interaction experiment in lymphatic branching between *Sema3f/g* and *PlexinA1/A2*. Of note, a very recent report demonstrates that full-length Semaphorin-3C functions as an inhibitor of tumor lymphangiogenesis and metastasis (Mumblat et al., 2015). Since we found the unique expression of SEMA3C in the limb (Fig. 2A), lymphatic vessel development in *Sema3c* mutants needs to be investigated. The mechanisms underlying the functional differences of SEMA3s in lymphatic vessel network formation will provide interesting topics for further study.

Defective lymphatic phenotypes of *Sema3f;Sema3g* double mutants and *Nrp2* mutants are restored during later embryonic stage (E17.5∼) or postnatal stage, and no apparent defective lymphatic network and valve formation was detectable in the adult skin. The restoration of defective lymphatic vessels has been observed in *Nrp2* mutants (Yuan et al., 2002) and in transgenic mice overexpressing a soluble VEGFR3 (Makinen et al., 2001). One possible explanation is that other SEMA3s such as SEMA3A, SEMA3B, SEMA3C, SEMA3D and/or SEMA3E may compensate for the lymphatic defects in *Sema3f;Sema3g* double mutants during later embryonic stage or postnatal stage. Indeed, SEMA3A/PlexinA1/NRP1 signaling is important for lymphatic valves formation (Bouvree et al., 2012; Jurisic et al., 2012). SEMA3E directly binds to PlexinD1 without neuropilins (Oh and Gu, 2013) and the expression of PlexinD1 was significantly detectable in dermal LECs (Fig. 5A). Although mechanisms regulating the restoration in these mutants remain unclear, our findings that SEMA3F and SEMA3G negatively regulate lymphatic branching and LEC growth provide important insights in understanding the process of lymphangiogenesis during development and pathological conditions such as metastatic cancer. A very recent study by the Gutkind's group has demonstrated that (1) loss of SEMA3F is a frequent event in head and neck squamous cell carcinomas (HNSCC), which correlates with increased tumor vascularity and metastasis, (2) SEMA3F requires NRP2 to promote LEC collapse, and (3) SEMA3F re-expression diminishes lymphangiogenesis and lymph node metastasis (Doci et al., 2015). Combined, SEMA3F/NRP2 signaling represents an important regulatory axis in tumor lymphangiogenesis, thus providing new therapeutic targets to halt aggressive metastasis.

## MATERIALS AND METHODS

### Experimental animals

*Nrp2* mice (Walz et al., 2002) were purchased from Jackson Laboratory. *Sema3g* mice were generated by the trans-NIH Knock-Out Mouse Project (KOMP). Genotyping for the *Sema3g-lacZ* allele was carried out with the following primer pairs: 5′-GCGGCTCTCCTACAGAGGTACTG-3′ and 5′-TGTCCTAGAGCCACGGACATTC-3′ as primers to detect wild-type allele; 5′-TAGCTGCACGGGCATTGAGC-3′ and 5′-GTCTGTCCTAGCTTCCTCACTG-3′ as primers to detect mutant allele. The *plexinA1*, *plexinA2,* and *Sema3f* mice have been reported elsewhere (Sahay et al., 2003; Suto et al., 2007; Yoshida et al., 2006). All animals and procedures for mouse experiments were approved by the National Heart, Lung, and Blood Institute (NHLBI) Animal Care and Use Committee.

### RNA *in situ* hybridization analysis

*In situ* hybridization using digoxigenin (DIG)-labeled riboprobes was essentially as previously described (Mukouyama et al., 2005, 2002). The sections (16 µm thickness) were permeabilized by 0.1% Tween20 in phosphate buffered saline (PBS) twice for 5 min each and then fixed with 4% paraformaldehyde (PFA, Electron Microscopy Science) for 30 min. The sections were washed with 0.1% Tween20 in PBS and incubated with 0.2% DEPC in PBS for 5 min. The sections were hybridized with anti-sense probe at 65°C for 16 h. The DIG-labeled cRNA probes diluted in hybridization solution (50% formamide, 5× SSC, 1% SDS, 50 µg/ml yeast RNA and 50 µg/ml heparin). After hybridization, the sections were washed in 50% formamide, 5× SSC and 1% SDS at 65°C for 30 min and then washed twice in 50% formamide and 2× SSC at 65°C for 45 min each. The sections were washed twice with 0.1% Triton X-100 in PBS for 5 min each at room temperature and then incubated with blocking buffer (0.1% Triton X-100 and 2% sheep serum in PBS) for 40 min. For DIG detection, the sections were incubated with alkaline phosphatase-conjugated anti-digoxygenin antibody (Roche 1:1000) for 3 h at room temperature. The hybridization signal was detected by BCIP/NTB (Roche). The probes for *Sema3c* and *Sema3f* were a generous gift from Y. Yoshida (Cincinnati, OH, USA; Yoshida et al., 2006). We have confirmed the specific *in situ* hybridization signals using anti-sense and sense cRNA probes.

### Immunohistochemistry

Whole-mount staining was performed as previously described (Li and Mukouyama, 2011; Mukouyama et al., 2002). The embryos were fixed by 4% PFA 4°C overnight. The forelimbs were dissected from embryos (E15.5, E17.5) and the skin peeled off; upper back skin was also peeled off. The samples were stained in the blocking buffer (0.1% TX-100 and 10% goat serum or donkey serum in PBS) containing primary antibodies overnight at 4°C. The primary antibodies used were: anti-PECAM1 antibody (rat monoclonal, clone MEC13.3, BD Pharmingen, 1:300) to detect endothelial cells; anti-LYVE1 antibodies (rabbit polyclonal, Abcam, ab14917, 1:500 or rat monoclonal antibody, clone ALY7, MBL, 1:500) and anti-PROX1 antibody (goat polyclonal, R&D, AF2727, 1:500) to detect lymphatic endothelial cells; anti-GFP antibody (rabbit polyclonal, with or without Alexa Fluor-488 conjugation, Invitrogen, 1:500) to detect GFP expression; anti-β-galactosidase (β-gal) antibody (rabbit polyclonal, Cappel, 55976, 1:5000) to detect β-gal expression; anti-phospho-Histon H3 (pSer28, rat monoclonal antibody, clone HTA28, Sigma, 1:1000) to detect proliferating cells. The samples were then washed 3 times in 0.1% TX-100 and 2% goat or normal donkey serum in PBS for 10 min each at room temperature. The samples were incubated with blocking buffer containing secondary antibodies for 5 h at 4°C. Different combinations of Alexa Fluor-488-, Alexa Fluor-568-, Cy3- or Alexa Fluor-647-conjugated secondary antibodies (Invitrogen 1:500 or Jackson Immunoresearch, 1:500) were used for different staining. The stained forelimb skins were mounted on slides, inner surface to the slide, in an anti-fade mounting media (Prolong Gold, Life Technologies). All confocal microscopy was carried out on a Leica TCS SP5 confocal (Leica).

Staining for cell culture was performed as previously described (Li and Mukouyama, 2011; Mukouyama et al., 2002). The cells were fixed with 4% paraformaldehyde (PFA)/PBS and stained with Alexa Fluor-568-conjugated Phalloidin (Invitrogen, 1:300) to detect actin cytoskeleton; TO-PRO-3 (Invitrogen, 1:2000) to detect nuclei; Ki67 (rabbit polyclonal, Vector Labs, VP-K451, 1:1000) to detect proliferating cells; anti-PROX1 antibody (goat polyclonal, R&D, AF2727, 1:500). Cell area measurement was performed by ImageJ software.

### Cell culture

Dermal-derived human lymphatic microvascular ECs (HMVEC-dLy-Neo, Lonza) were cultured in EBM-2 medium with growth supplements. About 75% of HMVEC-dLy-Neo cells are positive for PROX1 (data not shown). For cell contraction assays, 5×10^3^ of HMVEC-dLy cells were grown for over night on coverslips (Thermo Scientific) with fibronectin coating, then treated with 1 nM of alkaline phosphatase (AP) control protein, mouse AP-Sema3F with cleavage resistant point mutations (R580A/R582A), or mouse AP-SEMA3G for 30 min. Sprouting assays were modified as previously reported (Korff and Augustin, 1999). To make aggregates of HMVEC-dLy-Neo cells, 2000 cells were cultured in each well of ultra-low attachment round bottom 96 well plate (Corning Coster) for over night, then the cell aggregates were transferred into collagen gels. After collagen solidified, the cell aggregates were cultured with a basal EBM-2 medium (no serum and supplements) with 100 ng/ml VEGFC (Peprotech) and 0.5 nM of AP protein, 1 nM of AP-Sema3F (R580A/R582A) or 0.5 nM of AP-Sema3G, for 24 h. For proliferation assays, 1×10^4^ of the cells were cultured on coverslips for 5 h in a growth medium. After 5 h, the cells were cultured in the basal EBM-2 medium with 100 ng/ml VEGFC and 0.5 nM AP protein, AP-Sema3F (R580A/R582A) or AP-Sema3G, for 3 days. RNAi experiment of HMVEC-dLy-Neo, 5×10^5^ of the cells were cultured on 6 cm dish for 2 days. Then, siRNA (Ambion) were transfected 2 times by lipofectamine RNAiMAX (Life Technologies) each 2 days. The siRNAs for *Nrp2* and negative control are obtained from Silencer Select Validated/Pre-designed siRNA (Ambion); *Nrp2*#1 (ID#s16840), *Nrp2*#2 (ID#s16841) and Silencer Select Negative Control No.1 (cat#4390843). *Nrp2* knockdown was detected by immunoblot analysis with anti-NRP2 antibody (rabbit polyclonal, Santa Cruz, sc-5542, 1:500) and anti-β-actin antibody (mouse monoclonal, Sigma, clone AC-74, 1:5000).

### LECs isolation by FACS and RT-PCR

FACS isolation of LECs from embryos (James et al., 2013), and subsequent RNA isolation and RT-PCR was performed as previously described (Iwashita et al., 2003; James et al., 2013; Molofsky et al., 2003). RT-PCR primers sequence for mouse *Nrp2*, *Vegfr3* and *Plexins* were from PrimerBank (Spandidos et al., 2008, 2010; Wang and Seed, 2003): 5′-GACTTCATTGAGATTCGGGATGG-3′ and 5′-AACTTGATGTATAACACGGAGCC-3′ as primers for *Nrp2* (ID#116686133c2); 5′-GGCAAATGGTTACTCCATGACC-3′ and 5′-ACAACCCGTGTGTCTTCACTG as primers for *Vegfr3* (ID#226874801c1); 5′-GGTTGGACGATCTCTTCAAGC-3′ and 5′-GGGGAAGTATTCTGATTTGCCAT-3′ as primers for *PlexinA1* (ID#253683485c3); 5′-ACTCTGAGAATCGTGACTGGA-3′ and 5′-TGTTGGTGAGTGTAAGCACTTC-3′ as primers for *PlexinA2* (ID#113722112c2); 5′-CAGATACCACTCTGACTCACCT-3′ and 5′-GGCCCGTAGCTCAGTTAGG-3′ as primers for *PlexinA3* (ID#6679391a1); 5′-ACAGGGCACATTTATTTGGGG-3′ and 5′-CACTTGGGGTTGTCCTCATCT-3′ as primers for *PlexinA4* (ID#171543898c1); 5′-GAGCAAGCGCAACATACAGC-3′ and 5′-GGCTACAGTCAGCACTCGC-3′ as primers for *PlexinD1* (ID#153792703c2).
